# Predictability of epidemic malaria under non-stationary conditions with process-based models combining epidemiological updates and climate variability

**DOI:** 10.1186/s12936-015-0937-3

**Published:** 2015-10-26

**Authors:** Manojit Roy, Menno Bouma, Ramesh C. Dhiman, Mercedes Pascual

**Affiliations:** Howard Hughes Medical Institute, University of Michigan, Ann Arbor, 48109 MI USA; Department of Ecology and Evolutionary Biology, University of Michigan, Ann Arbor, MI USA; Faculty of Public Health and Policy, London School of Hygiene and Tropical Medicine, London, UK; Climate Dynamics and Impacts Unit, Institut Catala de Sciencies del Clima, 08005 Barcelona, Catalonia Spain; National Institute of Malaria Research (ICMR), Delhi, India; Department of Ecology and Evolution, University of Chicago, 1101 E 57th Street, Chicago, IL 60637 USA

**Keywords:** Epidemic malaria, India, Transmission model, Forecasting, Prediction skill

## Abstract

**Background:**

Previous studies have demonstrated the feasibility of early-warning systems for epidemic malaria informed by climate variability. Whereas modelling approaches typically assume stationary conditions, epidemiological systems are characterized by changes in intervention measures over time, at scales typically longer than inter-epidemic periods. These trends in control efforts preclude simple application of early-warning systems validated by retrospective surveillance data; their effects are also difficult to distinguish from those of climate variability itself.

**Methods:**

Rainfall-driven transmission models for falciparum and vivax malaria are fitted to long-term retrospective surveillance data from four districts in northwest India. Maximum-likelihood estimates (MLEs) of model parameters are obtained for each district via a recently introduced iterated filtering method for partially observed Markov processes. The resulting MLE model is then used to generate simulated yearly forecasts in two different ways, and these forecasts are compared with more recent (out-of-fit) data. In the first approach, initial conditions for generating the predictions are repeatedly updated on a yearly basis, based on the new epidemiological data and the inference method that naturally lends itself to this purpose, given its time-sequential application. In the second approach, the transmission parameters themselves are also updated by refitting the model over a moving window of time.

**Results:**

Application of these two approaches to examine the predictability of epidemic malaria in the different districts reveals differences in the effectiveness of intervention for the two parasites, and illustrates how the ‘failure’ of predictions can be informative to evaluate and quantify the effect of control efforts in the context of climate variability. The first approach performs adequately, and sometimes even better than the second one, when the climate remains the major driver of malaria dynamics, as found for *Plasmodium vivax* for which an effective clinical intervention is lacking. The second approach offers more skillful forecasts when the dynamics shift over time, as is the case of *Plasmodium falciparum* in recent years with declining incidence under improved control.

**Conclusions:**

Predictive systems for infectious diseases such as malaria, based on process-based models and climate variables, can be informative and applicable under non-stationary conditions.

**Electronic supplementary material:**

The online version of this article (doi:10.1186/s12936-015-0937-3) contains supplementary material, which is available to authorized users.

## Background

Millions of people living in the highlands and desert fringes around the tropics in Africa, Asia and South America are affected by seasonal and epidemic malaria, which occurs in areas of marginal environmental conditions for the development of the parasite and the population dynamics of the *Anopheles* mosquito vector. It is in these regions, where either local rainfall or temperature limit the population growth of the vector and the parasite’s development within the vector, that climate variability has the highest potential to strongly impact disease dynamics [1–5]. Thus, climate variability is fundamental to early-warning systems, and so are the consequences of longer-term trends in climate [5–8]. The ability to forecast and identify epidemic events is important to timely implementation of effective control policies, as recognized by efforts to develop malaria early-warning systems (MEWS) [9, 10]. In addition, process-based models that incorporate climate provide a basis for quantitatively evaluating the impact and effectiveness of intervention in the context of climate variability [11]. Given the inter-annual variability of rainfall and temperature, it is otherwise a challenge to determine whether any apparent fall (or rise) in disease incidence differs from what would be expected due to changes in the climate variables themselves, rather than in control efforts [12].

Extensive epidemiological and meteorological records spanning the past two decades in desert and semi-arid regions of northwest India provide an opportunity to further test predictive process-based models of epidemic malaria across multiple districts and for the two parasites, *Plasmodium falciparum* and *Plasmodium vivax*. Progress has recently been made with mathematical models that combine epidemiological processes and seasonal rainfall to represent the transmission dynamics of both falciparum and vivax malaria in semi-arid districts of India, validated by long-term surveillance data for the Kutch district in the state of Gujarat [3, 4, 13]. The parameterization of these models from surveillance records relies on recently developed inference methods for time series data [14, 15]. Investigation of the predictability of these models with ‘out-of-fit’ data remains unexplored however, especially under the changing conditions of recently intensified intervention.

Any prediction obtained from fitting such models to retrospective long-term data is expected to be useful if the dynamics of the system do not change much over time. In recent decades, however, improved socio-economic conditions and disease control policies that are revised every few years [16, 17], are often reflected in the shifting patterns of incidence, and these multiyear non-stationarities appear as a challenge to predictability. In particular, rapid diagnostic kits in hard-core malarious areas and indoor residual spraying (IRS) for controlling adult indoor resting mosquitoes, were adopted as national policies in India in 2005. Artemisinin-based combination therapy (ACT) was also introduced that year for treating falciparum malaria in high-risk areas. The subsequent policy revision in 2010 recommended universal use of ACT against *P. falciparum*, and complete treatment of confirmed malaria cases in place of presumptive treatment while waiting for confirmation of diagnosis. To a large degree, these measures have been effective in bringing down the incidence of falciparum malaria in subsequent years.  Besides control itself, other drivers of malaria non-stationarity in arid Northwest India include land-use change associated with irrigation projects, which can enhance the availability of breeding sites for the vector but also provide additional wealth, and therefore increase or decrease disease risk depending on temporal scale [18, 19].

 Anticipating changes in incidence patterns is difficult for models that do not explicitly include control efforts and/or trends in socio-economic conditions, and therefore, for systems whose overall intervention efforts are not easily available or quantifiable over time.  In addition, climate variability too operates over multiple time scales and introduces trends in disease incidence that confounds the evaluation of intervention programmes over multiple years.

In this paper, previously developed transmission models for falciparum and vivax malaria [3, 4] are used to obtain maximum likelihood estimates (MLE) of model parameters by fitting them to long-term surveillance data from four districts in northwest India. The resulting MLE model is then used to generate forward simulations that are compared with more recent (out-of-fit) data. Two issues are investigated regarding model prediction in the context of non-stationary transmission dynamics: (1) distinguishing between the respective roles of (inter-annual) climate variability and intervention methods in generating these non-stationarities, in particular the recent declining trends in incidence; and (2) improving predictability in the presence of such shifting conditions. These questions are addressed by generating model simulations with two different forecasting approaches. In the first one, the estimated initial conditions for the forward simulations are updated by incorporating the most recent data using a particle filtering technique. This procedure substantially improves the model’s forecasting ability, compared to simply simulating forward starting with the estimated initial conditions at the beginning of the record. Because the models explicitly include a climate driver (rainfall) but not control efforts, deviations between the simulation and the out-of-fit data can be used to quantify the impact of recent intervention measures in the context of climate variability. In the second approach, transmission parameters are also updated by refining the fit of the model over the most recent data in a moving window of time. Updating parameters on such a sliding timetable can sequentially adapt the model to the data and potentially improve its predictability as underlying socio-economic conditions, or intervention policies and efforts, change over time. In conclusion, the relative merits and limitations of these two methods are discussed, together with what the findings reveal about the current population dynamics of the two parasites in response to control and in the context of rainfall variability in the region.

## Methods

### Data

**Fig. 1:**
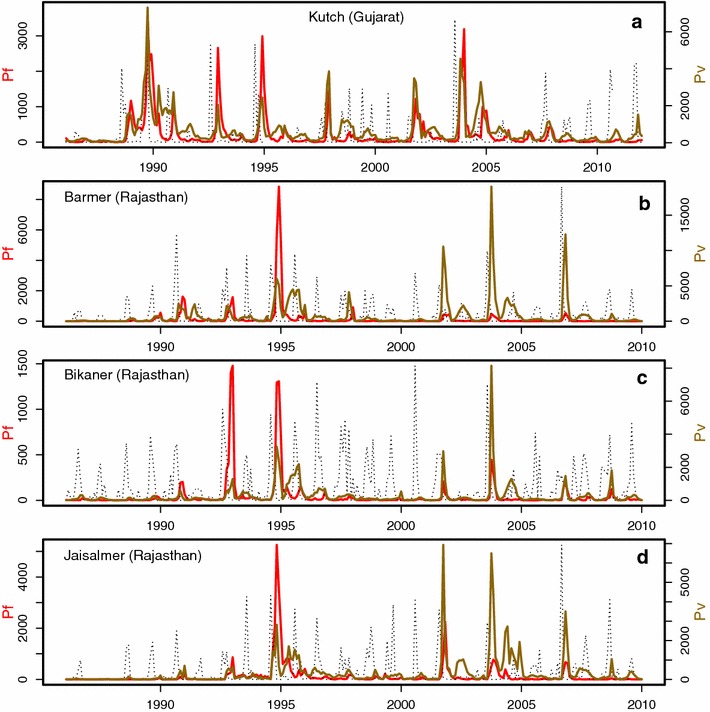
Malaria case data. **a**–**d** Monthly case data for *P. falciparum* (in *red*) and *P. vivax* (in *brown*, *right vertical axis*) are shown for Kutch, Barmer, Bikaner and Jaisalmer districts, respectively, along with the corresponding rainfall time series (in *dashed line*, rescaled to fit in the plot window; see Additional file 2 for the rainfall time series in their actual scale). The data for Kutch span January 1986–December 2011, and those for the other districts span January 1986–December 2009. All data exhibit strong seasonal patterns driven by monsoon rainfall, as well as interannual variability

The malaria data were obtained from the surveillance records for the district of Kutch in the state of Gujarat, and for those of Barmer, Bikaner and Jaisalmer in the state of Rajasthan. These four districts are located in the arid desert fringes of northwest India. The data, provided by the National Vector Borne Disease Control Programmes (in Rajasthan and Gujarat) through the National Institute of Malaria Research (in India), consist of monthly confirmed *P. falciparum* and *P. vivax* cases (from blood slides of patients visiting public health services and active surveillance) from January 1986 to December 2011 for Kutch, and from January 1986 to December 2009 for Barmer, Bikaner and Jaisalmer. (Hereafter, the two parasites are referred to as Pf and Pv respectively). Because of marginal environmental conditions for malaria transmission resulting from the arid climate in the region (see Additional file 1), yearly and inter-annual variability in rainfall generates pronounced variation in the size of seasonal epidemics (Fig. 1), as described in [3, 4]. Monthly rainfall data were recorded at local weather stations in each district, and supplied by the Indian Meteorological Department in Pune (India). Yearly population data were obtained via interpolation of the decadal census data between 1991 and 2011.

### Models and method

**Fig. 2:**
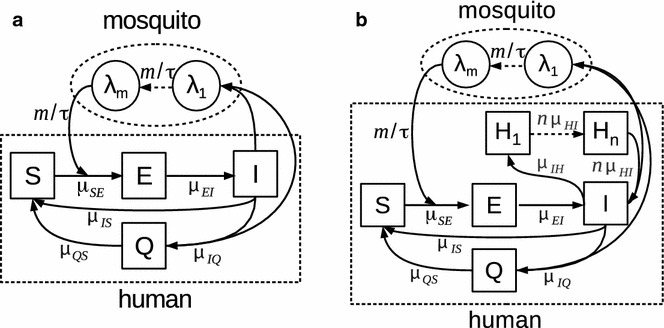
Malaria transmission models. **a**, **b** Model diagram for the population dynamics of falciparum and vivax malaria are respectively shown. Compartments for the human population are represented as *squares*, and those related to the force-of-infection via the mosquito vector (classes *λ*_1…*m*_ for *m* = 2) are represented as *circles*, with arrows indicating the direction of transition between classes. The per-capita rate of transition is included next to each *arrow* (see Additional file 3 for model equations and description)

The human component of the human-mosquito transmission model, for both Pf and Pv, is formulated by dividing the population into classes based on infection status as follows: S, for naive individuals who are susceptible to both infection and disease; E, for exposed or inoculated humans who are not yet infectious; I, for infectious humans that can transmit the pathogen to the vector; and Q, for individuals that have acquired some degree of immunity from disease and are therefore asymptomatic but still weakly infectious (Fig. 2). In a previous formulation the Q class was represented by two different classes to differentiate between individuals who are infectious and those who have recovered but are protected from clinical malaria upon re-infection [3]. That loop in the model keeps individuals away from contributing to reported cases while effectively implementing a reservoir of infection. Consideration of a single class, to account for a reservoir with a memory of patterns of infection in the recent past, provided a simpler and still accurate model, given the problem of parameter identifiability for processes associated with state variables (number of individuals in the corresponding classes) that remain unobserved [4]. The Pv model (Fig. 2b) additionally incorporates the relapse mechanism via a chain of *n*(=3) dormant classes H_1…*n*_ for humans who carry liver-stage hypnozoites [4]. Emergence of individuals from this chain into the I class generates infections in the absence of vector transmission and also primes the system for transmission earlier in the next rainy season than for Pf .

Given the absence of mosquito data, the vector dynamics are incorporated implicitly by a chain of multiple classes (two classes are used here, see Fig. 2) that effectively implement a distributed lag time between the fraction of the infected human population and the force of infection experienced by a susceptible individual. Technically, this lag is Gamma-distributed with mean *τ*, and it accounts for the developmental delay of malaria parasites within surviving mosquitoes. Variability in the dynamics that is driven exogenously arises from fluctuations in mosquito abundance and behavior, and is incorporated with three components: seasonality, rainfall (as a climate covariate), and environmental noise. Seasonal rainfall, as a climate driver of malaria, is known to have a complex relationship with the population dynamics of the disease [20] (see Additional file 2 for plots of monthly rainfall in their actual scales; fig. 1 shows these plots in relative scale).  For the region under study and following previous analyses [3, 4], a cumulative rainfall function was constructed from the original monthly data by aggregating precipitation over the preceding 5 and 4 months respectively for the Pf and Pv model. Additionally, for the Pf model, a threshold amount of 200mm was subtracted from the cumulative rain, and the resulting time series was used as the climate covariate (see supplement of [3, 4] for more details).

Each transmission model is formally related to the data by assuming that only a fraction *ρ* of new infections are detected by the surveillance methods, and introducing a measurement model that transforms these new infections into observed cases with a negative binomial distribution (see Additional file 3). The reporting fraction *ρ* was estimated along with other model parameters as described below.

To estimate model parameters, likelihood-based inference was carried out via a recently introduced iterated filtering method for calculating MLEs [3, 4, 13–15]. The method consists of executing two nested loops, with the outer loop essentially iterating an inner, ‘filtering’ loop, and in so doing generating a new, and improved, estimate of the parameter values at each iteration. The filtering loop is sequential in time (along the time series); it implements a selection process for a large number of ‘particles’ over time, via a sequential Monte Carlo filter. For each time step, a particle can be seen as a one-step simulation characterized by its own set of parameters that perform a random walk. Particles can survive or die as a result of a resampling process, with probabilities determined by their likelihood given the data (see supplement of [3], and [14, 15] for more details). The algorithm is known as ‘MIF’ and implemented in the R package ‘pomp’ [21]. This step-wise resampling serves to update the estimate of the parameters and state of the system (that is, the values of the state variables that are unmeasured) with the data up to the current time point. Thus, the resampling of particles (or ‘particle filtering’) over time can be used to update the estimated initial states of the system for forward simulation. When used together with the outer loop (‘iterated filtering’), estimates of the parameters are also updated by the data.

To assess the forecasting abilities of the model, each model for Pf and Pv was fitted to the early portion of the data, between January 1986 and August 2004 (18+ years), to obtain the MLE parameters, and simulated projections with the resulting MLE models were compared to the remainder of the data (out-of-fit data). The simulation procedure was carried out in two different ways:

1. In the first method, the original MLE model was used with updated initial states (particles) at August of each year between 2004 and 2008 (2010 for Kutch), and simulated forward over the next 12 months (September–August). Departures between the yearly projections and the out-of-fit data can be used to evaluate the impact of recent intervention efforts in the context of the observed multiyear rainfall variability.
2. In the second method, the model was refitted to the data over a moving time window of 4 years, starting with the window of September 2000–August 2004 (and shifting to the right by 1 year at a time). The resulting (updated) parameters, together with the updated initial states at August of each year, were then used to simulate forward over the next 12 months as before. This refitting procedure parameterizes non-stationarities in the data at the time scale of the sliding window. To focus on the changes in the transmission intensity, only a sub-set of the parameters directly related to the force of infection (the seasonality coefficients *b*_1...6_ and rainfall coefficient *b*_*r*_, see Additional file 3) were re-fitted, with the rest of the parameters held constant at their original MLE values.

The forecast method 2 is expected to be useful if the shifting patterns in the data are driven by changing control policies (with exceptions discussed below). On the other hand, when climate remains the primary driver of the dynamics, method 1 would suffice for prediction purposes and its success would be an indication that any trend is not the result of intervention. Choosing the month of August for the initial states and forecasting from the month of September is motivated by the major (post-monsoon) transmission season for malaria in India that usually occurs between September and December [3, 4].

A large number (typically 10,000) of simulations were generated with these two methods over the interval between September 2004 and December 2009 (2011 for Kutch), and monthly predictions were compared to the data over this interval. Using 3- and 5-year refitting windows for method 2 did not provide any additional advantage, so results are shown for a 4-year window. A time window shorter than 3 years does not have enough information in the data for the refitting to be useful, and a window longer than 5 years fails to capture short-term non-stationarities in the data. The choice of window length is further addressed in Discussion.

Hindcasting was used to examine two aspects of the fitted model’s prediction skill. First, a generalized *R*^2^ statistic was applied to measure forecasting skill, by comparing prediction error in total incidence (aggregated for the transmission season between September and December) between the process-based model and the simplest model of a ‘typical’ season given by the mean of the observed cases, with both errors normalized by the prediction variance of the former model. The prediction value *ŷ*_*t*_ was obtained by first accumulating the cases over a season (year *t*) for each of the simulations and computing the median for that given time from all these runs. The skill measure is defined as follows (see supplement of [3]):

1 $${\text{Skill}} = 1 - \frac{{{{\mathop \sum \nolimits_{{t = {\text{yr}}1}}^{{{\text{yr}}2}} \left( {y_{t} - \hat{y}_{t} } \right)^{2} } \mathord{\left/ {\vphantom {{\mathop \sum \nolimits_{{t = {\text{yr}}1}}^{{{\text{yr}}2}} \left( {y_{t} - \hat{y}_{t} } \right)^{2} } {v_{t} }}} \right. \kern-0pt} {v_{t} }}}}{{{{\mathop \sum \nolimits_{{t = {\text{yr}}1}}^{{{\text{yr}}2}} \left( {y_{t} - \mu } \right)^{2} } \mathord{\left/ {\vphantom {{\mathop \sum \nolimits_{{t = {\text{yr}}1}}^{{{\text{yr}}2}} \left( {y_{t} - \mu } \right)^{2} } {v_{t} }}} \right. \kern-0pt} {v_{t} }}}} \in \left[ { - \infty ,1} \right] ,$$

where *y*_*t*_ denotes observed cases, accumulated over September to December for the year *t*, *μ* is the mean of the observed cases over the years [yr1,yr2], and *v*_*t*_ is the prediction variance for year *t*. This variance weights the corresponding accuracy of the prediction in a given year by the precision of the model. Thus, years whose predictions are more precise (vary less) are given higher weights. This skill measure tells how much better the model’s ability to predict is compared to that of trivially using the mean: a positive value (≤1) indicates better prediction skill and a negative or zero value indicates poor skill.

Second, the model’s skill was evaluated in terms of the probability of predicting the occurrence of an epidemic ‘event’. An event is defined as the total number of cases in the fall months (September–December) of a given year exceeding a pre-specified threshold, such as their historical median in each district (two other event sizes, exceeding the historical 75th and 90th percentiles, are considered in Additional file 3). These thresholds represent the user’s definition of a large fall outbreak, and the model-predicted probability is computed over the 10,000 independent forecasts for each of the two methods described above. One can then assess the model’s skill as a binary classifier predicting whether or not a large outbreak would occur in a given year, for each of the future years (post-2004), in terms of the probability *p* exceeding a threshold *θ* (e.g., *p* > *θ* = 0.5), and then compare the resulting predicted outbreaks with the observed ones in the data. The accuracy of prediction over a time span of several years (both pre- and post-2004) is computed as a ratio of the total number of true positive (TP) and true negative (TN) events predicted by the model over these years, and the total number of observed positive (P) and negative (N) events during the same time:

2 $${\text{Accuracy}} = \frac{{{\text{TP}} + {\text{TN}}}}{{{\text{P}} + {\text{N}}}}\; \in \;[0,1]$$

A value close to or equal 1 indicates high accuracy of prediction.

## Results

**Fig. 3:**
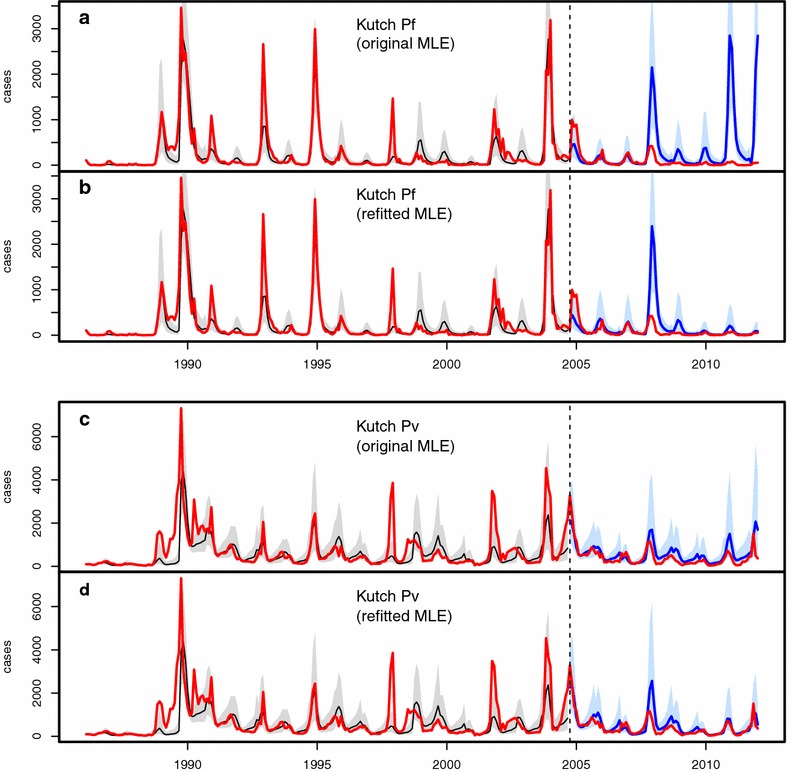
Comparison of model simulations with case data for Kutch. In all plots, monthly case data are shown in *red*, past simulations are in *black* (for the median of 10,000 simulations, with their corresponding 10–90 % confidence intervals, in *light grey*), and future simulations are in *blue* (10–90 % CI in *light blue*). **a**, **b** Present the results for falciparum malaria, and **c**, **d** for vivax malaria. Each *panel* consists of two plots, both including the same ‘past’ simulation but different future simulations corresponding respectively to those of method 1 (labelled ‘original MLE’) and method 2 (labelled ‘refitted MLE’)

**Fig. 4:**
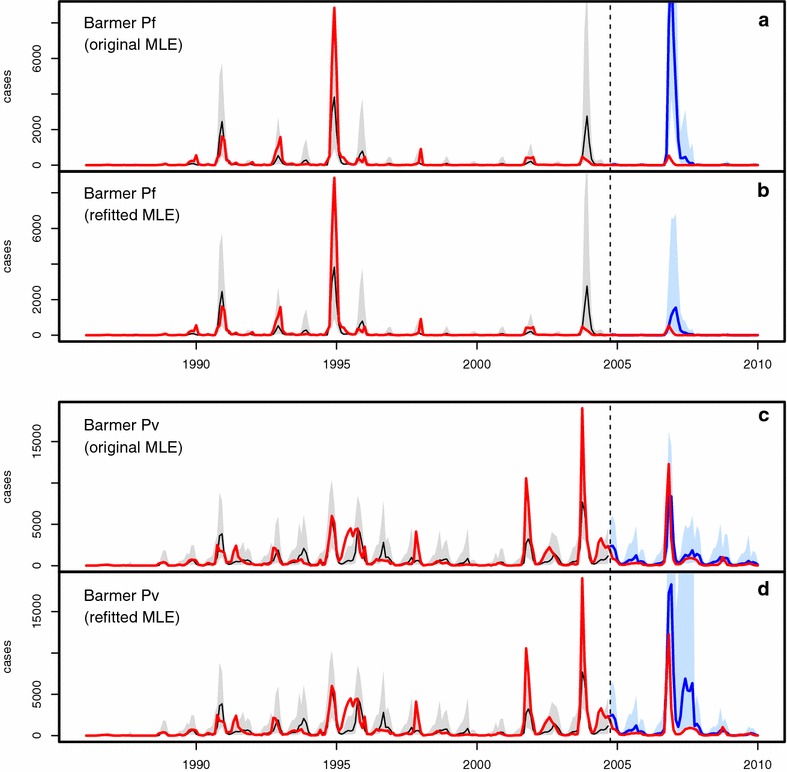
Comparison of model simulations with case data for Barmer. See the caption of Fig. 3 for description

**Fig. 5:**
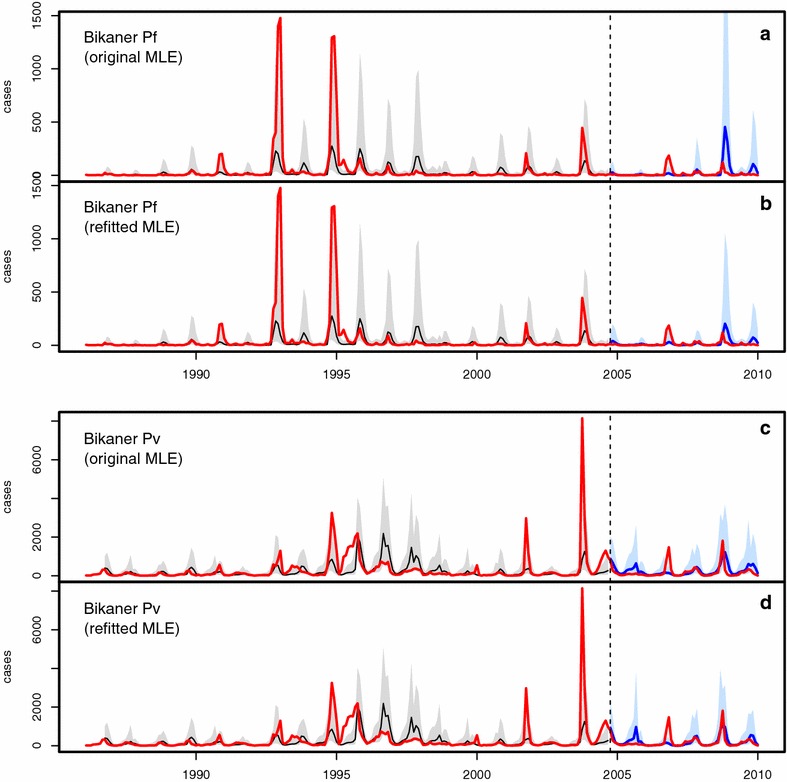
Comparison of model simulations with case data for Bikaner. See the caption of Fig. 3 for description

**Fig. 6:**
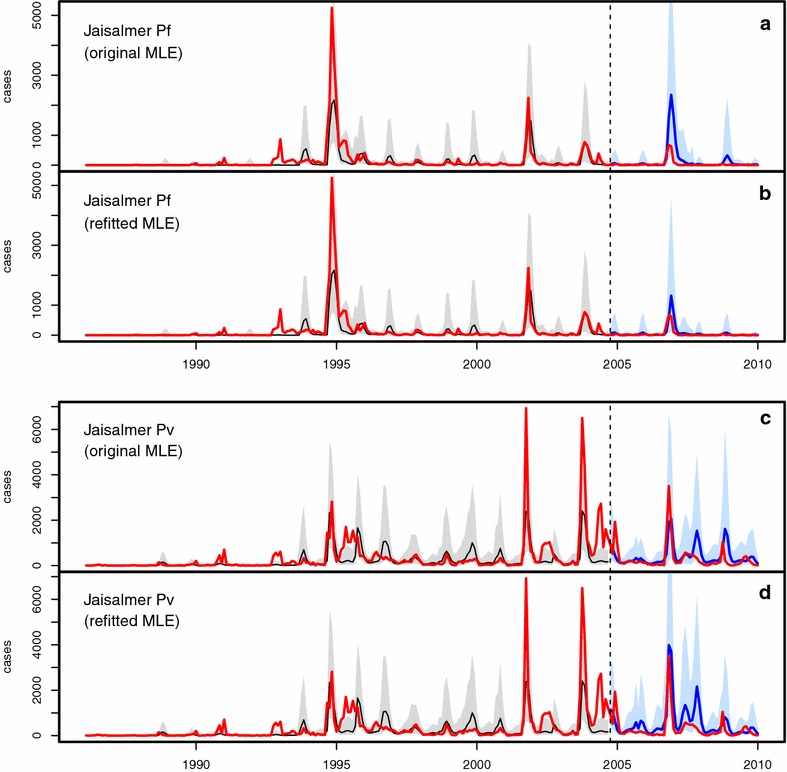
Comparison of model simulations with case data for Jaisalmer. See the caption of Fig. 3 for description

Figures 3, 4, 5 and 6 illustrate the comparisons between model predictions and data for Kutch, Barmer, Bikaner, and Jaisalmer, respectively. The top panel presents plots for Pf , and the bottom panel shows similar plots for Pv. In these plots, data are contrasted with both the ‘past’ (over January 1986–August 2004) and ‘future’ (September 2004 onwards) simulations. Past simulations are carried out with the original MLE model and sequential yearly updating of initial states at August of each year between 1986 and 2003 (as for method 1). The two future simulation plots in each panel are created using the two forecasting methods (see “Methods”) , with only updated initial states in the top ones (labelled ‘original MLE’ for method 1), and both updated initial states and parameters in the bottom ones (labelled ‘refitted MLE’ for method 2). The past simulation results are included to illustrate the hindcast performance of the models (see also [3]), and model predictability is evaluated here with the future simulations only, by comparison with the out-of-fit data. Hereafter the terms ‘future simulation’ and ‘forecast’ will be used interchangeably.

Comparing Pf data with the future simulations for the Kutch district (Fig. 3a, b, blue plots) illustrates the relative merits of the two methods. The data (in red) show a pronounced declining trend in incidence over this period, and observed cases are almost non-existent during the last 3 years between 2009 and 2011. Although this decline can be attributed to major changes in the national anti-malarial drug policy implemented during 2007-08, for example introducing ACTs for diagnosed Pf infections [16, 17], comparison to the forecasts can quantify the effect of intervention while taking into account the climate variability during that period. The forecast method 1, which only updates the initial states but not the model parameters, consistently overestimates the data over this interval, as expected from an effect of the new drug policy. The discrepancy between the out-of-fit data and the median simulation from the method 1 (Fig. 3a) is indicative of the effectiveness of these recent control measures against Pf in this region. By contrast, method 2, which updates both the initial states and parameters, and thus incorporates this improved control, agrees with the data quite well over the same time period, thereby substantially improving predictability despite this trend (Fig. 3b).

The Kutch Pv plots (Fig. 3c, d, blue plots) show a contrasting result, with predictions close to the observed case possibly for two reasons. First, it is well known that relapses often play a major role as a hidden reservoir in maintaining vivax malaria, especially in northwest India [4, 22], and thus the predictability of the vivax model may depend more on its relapse component (Fig. 2b, also see [4]), and less on factors that modify transmission intensity such as control efforts against the vector. Thus, the population dynamics of the disease are expected to be more resilient to control. Second, given the well-documented inadequacies of currently available treatments against relapses and more virulent strains [22–24], one would not expect a strong effect of such kinds of control (see “Discussion”). Method 2 is, therefore, unlikely to significantly improve the quality of prediction for vivax malaria over method 1, as found here.

The Barmer Pf data (Fig. 4a, b) exhibits a high degree of intermittency in the occurrence of large outbreaks, with mostly small outbreaks and occasional large flare-ups, for example in the fall of 1994. Fitting the models to such intermittent data can be difficult: for example, in year 2004 (in the past simulation), the MLE model generates a large fall outbreak driven by strong monsoon rain that year (see Fig. 1b), although the observed epidemic was small. Method 1 predicts an even larger outbreak in 2006, which is also the year that experienced the strongest monsoon over the entire time span (Fig. 1b), while method 2 however forecasts a much smaller outbreak that year. Both methods correctly predict the subsequent low incidence.

The Barmer Pv plots (Fig. 4c, d) highlight a different contrast between the forecast methodologies presented here. The forecast from method 1 already agrees with the data quite well (Fig. 4c, blue plot), which suggests the inadequacy of recent control efforts against vivax malaria, as mentioned above. Thus a correction of the model with method 2 is not needed, and can even cause a deviation from observations, biased by shorter patterns in a smaller window (such as the two large epidemics of 2001 and 2003) (Fig. 4d). The outcomes of both methods are presented here to illustrate their differences; in practice, the goal is not the comparison of the two methods *per se*, but the question of whether one should further refine the fit or not.

The Bikaner Pf plots (Fig. 5a, b), much like the Kutch plots discussed before (Fig. 3a, b), illustrate the relative superiority of method 2 over method 1, when application of the latter, and the data itself, both reveal apparent non-stationarities. For the Pv data though (Fig. 5c, d), as for those of Kutch earlier, the difference between the two methods is minimal.

The Jaisalmer Pf plots (Fig. 6a, b) are similar to the Bikaner ones described above, and the forecasting abilities of the two methods differ in a similar way. As for the Jaisalmer Pv plots (Fig. 6c, d), method 2 appears to generate better forecasts in the more recent years.

The MLE values of the estimated model parameters for all districts are consistent with estimates presented in previous studies for Pf and Pv malaria in Kutch [3, 4], which is expected given the similarities of the prevailing climatic and other environmental conditions.  In particular, the reporting fraction *ρ* is estimated to fall in the range of 1-3%, a low value that is consistent with a high degree of under-reporting [3, 4].

Table 1 presents the skill measure (expression 1) for the past period and for the two methods and the future simulations, for each of the four districts (see “Methods”). The high skill values in the past for both Pf and Pv are indicative of the quality of the model fitting to the retrospective data. Comparing the two future skills for each district shows they can be positive or negative, encompassing a wide range of performance. For example, for Kutch and Bikaner, the forecasts with method 2 show a much higher skill than those with method 1 in predicting Pf malaria, but perform only moderately better for Pv malaria, consistent with the findings of Fig. 3. For Barmer and Jaisalmer Pv, on the other hand, method 1 has a higher skill than method 2, also as expected. This shows that there is no reason to refine the fit when there is no apparent trend or systematic deviation of the predictions; this can overly weight recent patterns in an unnecessary way, as mentioned before.

**Table 1:** Skill measures

	Kutch		Barmer		Bikaner		Jaisalmer	
	Pf	Pv	Pf	Pv	Pf	Pv	Pf	Pv
Past	0.985	0.901	0.992	0.997	0.840	0.807	0.802	0.994
Future^(1)^	−2.027	0.567	0.653	0.926	−0.225	0.644	0.591	0.555
Future^(2)^	0.463	0.785	0.979	0.749	0.064	0.709	0.913	0.407

Values of prediction skill are shown for Kutch, Barmer, Bikaner and Jaisalmer

‘Past’ skill is computed for the years 1986–2003, and the two future skills are computed for the years 2004–2011 for Kutch, and 2004–2009 for the other districts

Future skills for the two forecast methods are labelled Future^(1)^ and Future^(2)^, respectively

Table 2 shows the accuracy of predicting whether or not a large fall outbreak will occur in a given year (see “Methods”), over the same past and future periods and four districts as in Table 1. In all cases, method 2 offers higher accuracy in predicting falciparum malaria, whereas method 1 is at least as accurate for vivax malaria if not better (in Bikaner), in agreement with the findings presented before. Similar results are found when the outbreak size is defined in terms of historical 75th and 90th percentile of observed fall cases (see Additional file 3).

**Table 2:** Prediction accuracy

	Kutch		Barmer		Bikaner		Jaisalmer	
	Pf	Pv	Pf	Pv	Pf	Pv	Pf	Pv
Past	0.778	0.722	0.722	0.778	0.833	0.778	0.778	0.722
Future^(1)^	0.5	0.875	0.833	1	0.5	0.667	0.5	0.667
Future^(2)^	0.875	0.875	1	0.833	0.667	0.667	0.833	0.667

Accuracy of predicting the occurrence of a large fall outbreak in a given year is shown for the four districts and past and future years (other details are the same as those in Table 1)

## Discussion

In regions that experience substantial climate variability, it is difficult to determine whether any particular change in the temporal patterns of malaria incidence results from intervention efforts or simply from the climate conditions *per se*. A further challenge arising from non-stationary temporal patterns is their lack of predictability based on models that are parameterized and validated with retrospective data. This paper addresses these two issues with a comparative assessment of two different forecasting approaches applied to climate-driven transmission models for falciparum and vivax malaria, and long-term surveillance data from four districts in northwest India. Both approaches make use of a particle filtering technique to update the estimated initial conditions of yearly forecasts based on the data up to that time, thus partially improving the model’s prediction skill. The second approach further updates the transmission parameters of the model by refitting them to the data over a moving time window. This additional updating procedure helps account for factors such as socio-economic development or improved control policies (not included in the model).

Results presented here indicate conditions under which assimilating data in these two ways can help improve forecasts and also be informative on the effect of interventions. In particular, the first approach provides the means to evaluate the impact of control efforts in the context of climate variability. An example of this application was shown for the district of Kutch where the model clearly overestimates the observed cases of Pf in recent years (Fig. 3a). By contrast, model predictions closely capture the observed cases of Pv in most districts, including Kutch. Thus rainfall variability *per se* can adequately explain the inter-annual variability in the size of seasonal outbreaks of Pv but not Pf. This difference can be accounted by the adoption of an effective drug treatment (ACT) for the latter parasite, whereas the use of an ineffective drug treatment combined with its ability to relapse, make the former more resilient to intervention. Control efforts targeting the vector such as IRS ( Insecticide Residual Spraying) also appear inadequate [25].

Thus the choice between the two methods depends on the particular knowledge and questions about the introduction of new intervention measures and recent changes in incidence.  When such changes are absent (or unknown), the first approach would be the method of choice.  When its application results in prediction failures (despite good retrospective skill), or when changes in control measures and policies are known to occur, a switch to the second method would be warranted for more reliable prediction.

One potential limitation of the refitting procedure is the somewhat arbitrary choice of the length of the moving time window used to update transmission parameters. As noted before, too short or too long a window, relative to the extent of the prevailing inter-annual variability in the data, is misleading for prediction purposes. It is unlikely however that analyses relying on retrospective records alone would allow one to identify an optimal time scale, except perhaps in simple situations. For example, the anti-malarial drug policy in India has been revised every few years in recent decades [17], which may suggest the use of a time scale that closely follows the evolution of such updates. However, other socio-economic developments, such as land-use practices related to irrigation and agriculture in arid regions of northwest India, also impact malaria trends at slower time scales [18, 19, 26]. In other (less complex) situations, it may be possible to extract a candidate time scale from the data itself, by examining the question of window size systematically using the retrospective data, in combination with power spectrum analyses that are localized in time, such as the wavelet spectra [18, 27, 28].

It is also apparent from the results that the recurrent updating of parameters (approach 2) works better in those districts whose non-stationarity consists of a decreasing rather than an increasing trend. This is because multiyear increases in the size of outbreaks tend to be local in time and often followed by a reversal, which leads to over-predicting malaria burden at the transition. One likely explanation is that local increases typically generate reactive intervention responses [29]. Although such cycles are possible when intervention relaxes after an apparent decrease in malaria burden, long-term trends reflecting socio-economic improvements and/or the adoption of a new intervention, against which the vector or the parasite have not yet evolved resistance, tend to be sustained at a longer scale and therefore are more easily corrected for.

The yearly predictions generated by either approach are produced by driving the model with actual climate variables (rainfall data). However, to be operationally useful, such predictions need to be generated for future times whose relevant climate data are not yet available. A default approach to handle this issue is to rely on a synthetic 12-month long climate series obtained by averaging the corresponding monthly values over previous years. In yearly forecasts from the end of the monsoons, this works well because most of the relevant rainfall has already been observed. This limits, however, the lead time of outbreak prediction. An alternative, and potentially better, strategy that would afford longer and more flexible lead times, would consist of first generating seasonal climate forecasts [2] and then using these to drive the transmission model. The accuracy of this approach would clearly depend on the accuracy of the climate forecasts themselves. In the same vein, the usefulness of the methods presented here strongly depends on the quality of the initial fit to the ‘training’ data set.

One simplification adopted in the model for both falciparum and vivax malaria is the consideration of a single partially immune class (Q), which ignores the details of an age-stratified immunity distribution, but still proves sufficient in capturing the transmission dynamics in this epidemic region.  Such formulation may potentially limit the scope of this modeling framework in areas with higher transmission intensity, where host immunity from repeated infection is likely to play a complex and more important role.

 Prediction with models that combine climate variability and epidemiology is likely to be successful in seasonal, low transmission regions, at the edge of the distribution of the disease, in arid regions and highlands [30].  It is also here that the temporal patterns of incidence are most variable at interannual time scales, and this variability can be exploited to inform the parameterization of the models from retrospective surveillance data.  Nevertheless, the extent to which one can extend this kind of forecasting beyond these fringe areas when additional epidemiological data is available, especially on the age distribution of cases, remains unexplored.  Clearly, for highly endemic areas where there is seasonality but very weak interannual variability in incidence, early-warning systems for predicting ‘outbreaks’ are no longer of interest.  There may still be variation however in the seasonal patterns themselves in response to climate variability as a recent study illustrates [31].

 The definition of outbreak size used to evaluate prediction accuracy/performance is by no means unique [32], and can potentially affect the outcome of the methods presented here.  The prediction accuracy was evaluated here by using different thresholds to define the occurrence of an outbreak when cases surpass a given value.  Such a threshold was specified to a priori mimic the situation of a choice based on a level of incidence the public health system considers of concern.  Alternatively, ROC (Receiver Operating Characteristics) curves could be used for this purpose [33].  Moreover, the skill measure itself does not rely on a threshold and uses the number of cases themselves; hence, it does not depend on the definition of an outbreak as a discrete event.

 It is certainly possible to devise more elaborate models of malaria than those used here, to account for detailed description of the processes underlying the dynamics in such a complex infection [29, 34]. In the absence of specific data on the levels of intervention and relevant socio-economic changes, these models do not solve the problem of non-stationary dynamics. Furthermore, these more complex models quickly become difficult, if not impossible, to parameterize and validate from time series data. In this regard, the simpler models used here provide an effective means of projecting the expected future course of the system given the information contained in retrospective temporal patterns. They are particularly useful to quantify deviations from this expectation, and therefore, detect the effect of intervention in a way that controls for climate variability, as discussed before.

The WHO’s Roll Back Malaria global strategic initiative stipulates a target for MEWS to detect 60 % of epidemics within 2 weeks [35]. For climate-based early warnings and forecasting systems, a major challenge is that of translating promising scientific studies of well-demonstrated climate-malaria relationships into operational predictive models [3]. As this study demonstrates, one approach towards this objective is to make better use of state-of-the-art statistical methodologies to ‘train’ these models with long-term surveillance data and to recurrently assimilate new data in systems that are unavoidably non-stationary.

## Additional files

10.1186/s12936-015-0937-3 A figure file illustrating the major climate zones of India, highlighting the arid and semi-arid climate conditions that span most of the states of Gujarat and Rajasthan in northwest India (adapted from the original source in http://besttofind.com/Img/india_climate_map.jpg).
10.1186/s12936-015-0937-3 A figure file showing the monthly rainfall time series data for the four districts in their actual scale (the same data are also shown in Fig. 1 in relative scale with the dashed line).
10.1186/s12936-015-0937-3 A text file describing the models for Pf and Pv together with the measurement model. The file also contains a supplementary table showing the values of accuracy for predicting the occurrence of a large fall outbreak in a given year for the four districts and for past and future years.
