# Supplementary material for: Predictability of epidemic malaria under non-stationary conditions with process-based models combining epidemiological updates and climate variability
Source: Malar J. 2015 Oct 26;14:419. doi: 10.1186/s12936-015-0937-3 (PMC4623260; doi:10.1186/s12936-015-0937-3)
Supplement: Supplementary file 1 — 10.1186/s12936-015-0937-3 A figure file illustrating the major climate zones of India, highlighting the arid and semi-arid climate conditions that span most of the states of Gujarat and Rajasthan in northwest India (adapted from the original source in http://besttofind.com/Img/india_climate_map.jpg). [file 12936_2015_937_MOESM1_ESM.docx]

**Supplementary Text**

The only difference between the transmission models for *P. falciparum* and *P. vivax* malaria is the presence of a chain of hypnozoite classes H_1..._*_n_* for humans in the latter (Fig.2). The objective of these population-level models is to capture the key aspects of human, parasite and vector dynamics, while remaining parsimonious enough for biologically relevant parameters to be estimated directly from available surveillance data on monthly cases over time [3,4].

The equations for the human component of the *P. vivax* model are follows:

$${dS}/{dt}=\left( \delta P+{dP}/{dt} \right)+\mu_{IS}I+\mu_{QS}Q-\mu_{SE}E-\delta S,$$

$${dE}/{dt}=\mu_{SE}E-\mu_{EI}E-\delta E,$$

${dI}/{dt}=\mu_{EI}E+n\mu_{HI}H_{n}-\left( \mu_{IS}+\mu_{IQ}+\mu_{IH} \right)I-\delta I,$ (S1)

$${dH_{1}}/{dt}=\mu_{IH}I-{n\mu}_{HI}H_{1}-\delta H_{1},$$

$${dH_{i}}/{dt}={n\mu}_{HI}H_{i-1}-{n\mu}_{HI}H_{i}-\delta H_{i}\left[ fori=2,\ldots,n \right],$$

$${dQ}/{dt}=\mu_{IQ}I-\mu_{QS}Q-\delta Q.$$

The *P. falciparum* model is nested within the *P. vivax* model, and the corresponding equations follow by setting $\mu_{HI}=0$ and removing the H_1..._*_n_* classes in Eqs.S1 above. The birth rate for the S class is set to ensure that $S\left( t \right)+E\left( t \right)+I\left( t \right)+Q\left( t \right)+\sum_{i} H_{i}\left( t \right)=P\left( t \right)$, where $P\left( t \right)$ is the population size of the said district at time *t* obtained from interpolated census data (see supplementary table S1 in [4] for parameter definitions).

The role of mosquitoes in the transmission from infected to susceptible humans is represented implicitly through their effect in generating a distributed time delay between the current rate of transmission experienced by a susceptible human at time *t*, $\mu_{SE}\left( t \right)$, and a chain of classes *λ*_1…_*_m_* representing the force of infection resulting from levels of infection in the human population at all previous times.

The equation for the measurement model, which couples the continuous-time dynamics of the model (Eqs.S1) with the discrete-time sequence *y*_1_,…,*y_N_* of monthly reported case data at times *t*_1_,…,*t_N_*, is given by

$y_{k} \sim\text{NegBin}\left( M_{k},\sigma_{\text{obs}}^{2} \right)$ for $M_{k}= \rho\int_{t_{k-1}}^{t_{k}} \left[ \mu_{EI}E\left( s \right)+n\mu_{HI}H_{n}\left( s \right) \right]ds$ , (S2)

where NegBin(*a*,*b*) is the negative binomial distribution with mean *a* and variance *a* + *a*^2^*b*. Further details of the description and implementation of the model can be found in the supplement of [4].

**Supplementary Table S1**

**A.**

|  | **Kutch** | | **Barmer** | | **Bikaner** | | **Jaisalmer** | |
| --- | --- | --- | --- | --- | --- | --- | --- | --- |
|  | Pf | Pv | Pf | Pv | Pf | Pv | Pf | Pv |
| Past | 0.833 | 0.889 | 0.944 | 1 | 0.833 | 0.778 | 0.778 | 0.778 |
| Future^(1)^ | 0.5 | 0.875 | 0.833 | 0.833 | 0.667 | 0.833 | 0.833 | 0.667 |
| Future^(2)^ | 0.75 | 0.875 | 0.833 | 0.833 | 0.667 | 0.667 | 1 | 0.667 |

**B.**

|  | **Kutch** | | **Barmer** | | **Bikaner** | | **Jaisalmer** | |
| --- | --- | --- | --- | --- | --- | --- | --- | --- |
|  | Pf | Pv | Pf | Pv | Pf | Pv | Pf | Pv |
| Past | 1 | 0.889 | 0.889 | 0.889 | 0.889 | 0.722 | 1 | 0.944 |
| Future^(1)^ | 0.625 | 1 | 0.833 | 1 | 1 | 1 | 0.833 | 1 |
| Future^(2)^ | 1 | 1 | 0.833 | 1 | 1 | 1 | 0.833 | 0.833 |

**Table S1. Prediction accuracy computed using expression (2)**

Values of accuracy for predicting the occurrence of a large fall outbreak in a given year are shown for the four districts and past and future years, where the outbreak size is historical 75^th^ percentile (table A) and 90^th^ percentile (table B) of observed fall cases (other details same as in Table 2).
