# Supplementary material for: Predictability of epidemic malaria under non-stationary conditions with process-based models combining epidemiological updates and climate variability
Source: Malar J. 2015 Oct 26;14:419. doi: 10.1186/s12936-015-0937-3 (PMC4623260; doi:10.1186/s12936-015-0937-3)
Supplement: Supplementary file 2 — 10.1186/s12936-015-0937-3 A figure file showing the monthly rainfall time series data for the four districts in their actual scale (the same data are also shown in Fig. 1 in relative scale with the dashed line). [file 12936_2015_937_MOESM2_ESM.docx]

**
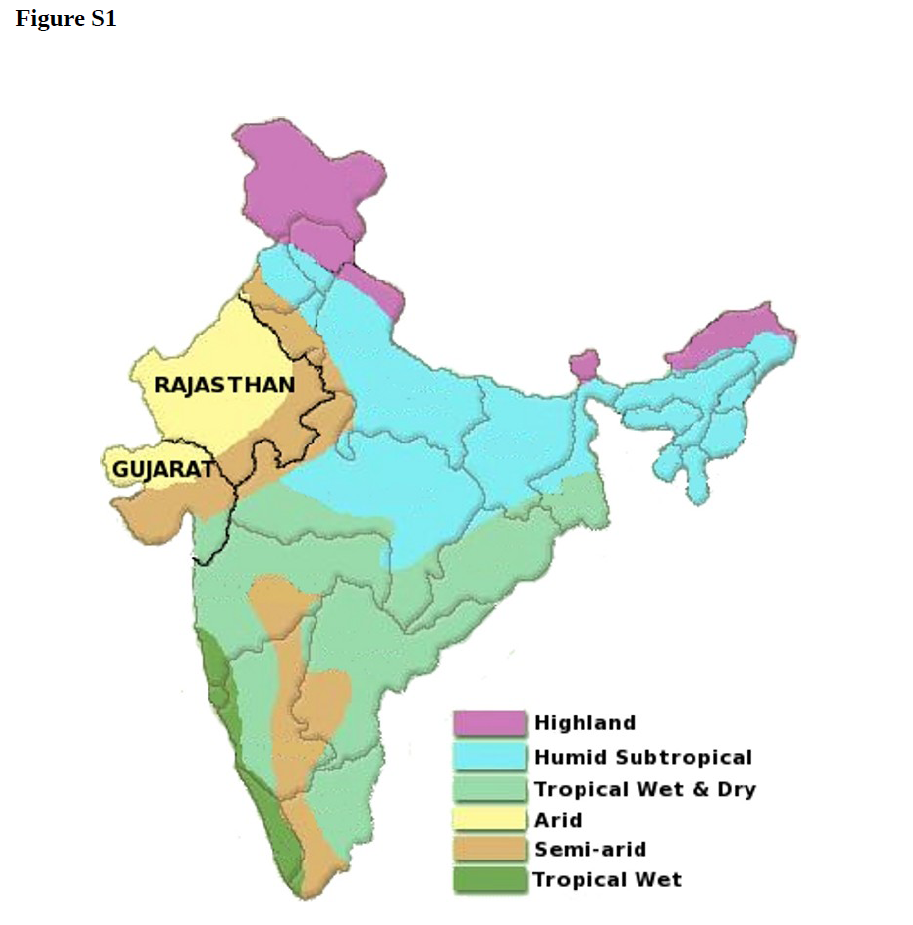
**

**Figure S1. Major climate regions of India**

The figure illustrates the major climate zones of India, highlighting the arid and semi-arid climate conditions that span most of the states of Gujarat and Rajasthan in NW India (adapted from the original source in <http://besttofind.com/Img/india_climate_map.jpg>).
