# Supplementary material for: Predictability of epidemic malaria under non-stationary conditions with process-based models combining epidemiological updates and climate variability
Source: Malar J. 2015 Oct 26;14:419. doi: 10.1186/s12936-015-0937-3 (PMC4623260; doi:10.1186/s12936-015-0937-3)
Supplement: Supplementary file 3 — 10.1186/s12936-015-0937-3 A text file describing the models for Pf and Pv together with the measurement model. The file also contains a supplementary table showing the values of accuracy for predicting the occurrence of a large fall outbreak in a given year for the four districts and for past and future years. [file 12936_2015_937_MOESM3_ESM.docx]

**
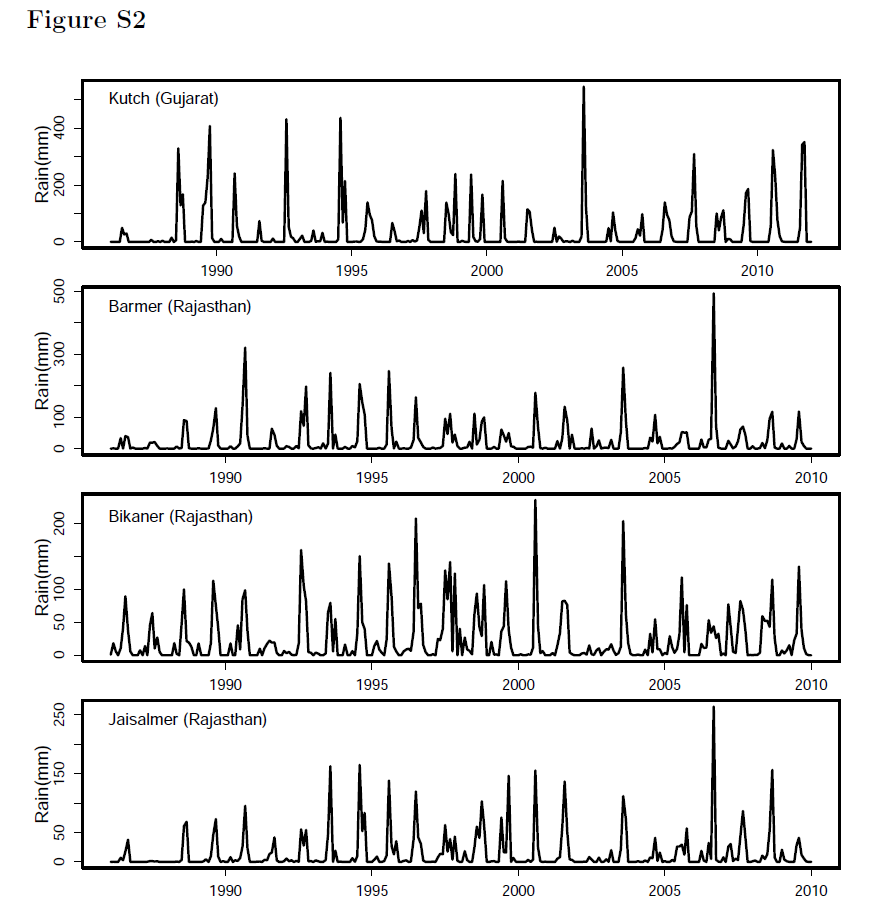
**

**Figure S2. Monthly rainfall time series**

Monthly rainfall time series data for the four districts are shown in their actual scale (the same data are also shown in fig.1 in relative scale by dashed line).
